# Long non-coding RNAs in cancer glycolysis and metabolism: mechanisms and translational opportunities

**DOI:** 10.1038/s41419-025-08289-2

**Published:** 2025-12-08

**Authors:** Yaru Ren, Ziyu Zhang, Xudong Lei, Lei Shi

**Affiliations:** 1 RNA Oncology Group, School of Public Health, Lanzhou University, Lanzhou, PR China; 2 Sun Yat-sen University Cancer Center Gansu Hospital, Lanzhou, PR China; 3 Cancer Research UK Manchester Institute, The University of Manchester, Manchester, UK

**Keywords:** Cancer metabolism, Prognostic markers

## Abstract

Long non-coding RNAs (lncRNAs) have emerged as critical regulators of cancer metabolism, particularly in the reprogramming of glycolysis that supports tumor growth and survival. Once considered non-functional genomic “noise”, lncRNAs influence metabolic adaptation by modulating glycolytic enzymes, transcription factors, and signaling pathways, while also shaping the tumor microenvironment through immune and stromal interactions. In addition, lncRNA-encoded micropeptides provide an extra layer of metabolic control, underscoring their functional diversity. These features indicate lncRNAs as promising diagnostic biomarkers and therapeutic targets, particularly in the context of personalized cancer treatments. RNA-based therapies demonstrate preclinical efficacy in targeting glycolytic lncRNA and reversing drug resistances. Nonetheless, challenges remain, including delivery specificity, off-target effects, and limited clinical validation. Advances in single-cell multi-omics, spatial transcriptomics, and artificial intelligence may offer new avenues to overcome these challenges. Collectively, lncRNAs represent both mechanistic drivers of glycolysis and promising targets for innovative diagnostic and therapeutic strategies in cancer.

## Facts

- Dysregulated lncRNAs modulate cancer glycolysis by regulating key glycolytic enzymes, glucose transporters, and oncogenic signaling pathways.
- The interplay between lncRNAs and post-translational modifications of glycolytic enzymes could provide insights into metabolic regulation.
- lncRNAs regulate the tumor microenvironment by influencing immune responses, fibroblast activation, extracellular matrix remodeling, and metabolic adaptation of tumor cells.
- Emerging technologies like single-cell multi-omics and spatial transcriptomics offer promising avenues to study the context-specific functions of lncRNAs in cancer metabolism.

## Introduction

Cancer is a complex disease characterized by uncontrolled cell growth, invasion, and metastasis [1]. Hanahan and Weinberg initially emphasized self-sufficiency in growth signals, insensitivity to anti-growth signals, tissue invasion & metastasis, sustained angiogenesis and evading apoptosis as essential hallmarks of cancer, and later expanded to include energy metabolism reprogramming and immune evasion [2, 3]. Metabolic reprogramming has now been recognized as a defining feature [4]. This field concept dates to the 1920s, when Otto Warburg first observed that cancer cells predominantly generate energy through anaerobic glycolysis, rather than through the citric acid cycle and oxidative phosphorylation used in normal cells [5]. Understanding cancer-specific glycolysis may therefore reveal novel therapeutic opportunities.

Non-coding RNAs (ncRNAs) are transcripts from the human genome that do not encode proteins. They encompass various types such as microRNA, lncRNA, circular RNA, and PIWI-interacting RNA [6]. lncRNAs, a subset of ncRNAs longer than 200 nucleotides, are transcribed by PoI II and typically undergo processing that includes 5′-end m7G caps, 3′-end poly(A) tails, and splicing [7]. Research over the past decade has transformed their perception from “junk noise” or “dark matter” to diverse functional molecules involved in various cellular processes, including RNA processing, transcription regulation, post-transcription regulation, translation regulation [8]. Emerging studies have showed that lncRNAs exhibit complex and precise regulatory characteristics in cancer, for example, acting as oncogenes or tumor suppressors [9].

The crosstalk between lncRNAs and glycolysis has been implicated in cancer progression. This review emphasizes on the fundamental roles of lncRNAs in cancer glycolysis, aiming to deepen our understanding of the modulatory networks, and provide a better theoretical foundation for clinical diagnosis and treatment of metabolic related cancer.

## The glycolysis in cancer

Glycolysis is a crucial metabolic pathway with a significant role in cancer metabolism. In normal cells, glycolysis occurs in the cytoplasm, where glucose is broken down into pyruvate, generating a small amount of ATP in the process. Under aerobic conditions, pyruvate is transported into the mitochondria for further oxidation via the citric acid cycle and oxidative phosphorylation, which generate the majority of ATP. In contrast, cancer cells exhibit a metabolic shift known as the “Warburg effect” or aerobic glycolysis. Despite the presence of sufficient oxygen, cancer cells preferentially convert glucose to lactate via glycolysis, which enables cancer cells to rapidly generate ATP, rather than metabolizing it in the mitochondria [10].

Glycolysis consists of two phases: the preparatory phase and the payoff phase. In the preparatory phase, one molecule of glucose undergoes sequential enzymatic reactions catalyzed by hexokinase (HK), phosphoglucose isomerase, phosphofructokinase (PFK), aldolase, and triosephosphate isomerase, resulting in the production of two molecules of glyceraldehyde 3-phosphate (G3P) and the consumption of two ATP molecules. In the payoff phase, G3P is further processed through reactions catalyzed by G3P dehydrogenase (GAPDH), phosphoglycerate kinase (PGK), phosphoglycerate mutase (PGAM), and pyruvate kinase (PK), leading to production of pyruvate and ATP [11]. Targeting glycolytic enzymes presents a promising approach in cancer therapy, particularly when combines with other treatments to exploit the metabolic vulnerabilities of cancer cells.

## The molecular functions of lncRNAs

**Fig. 1:**
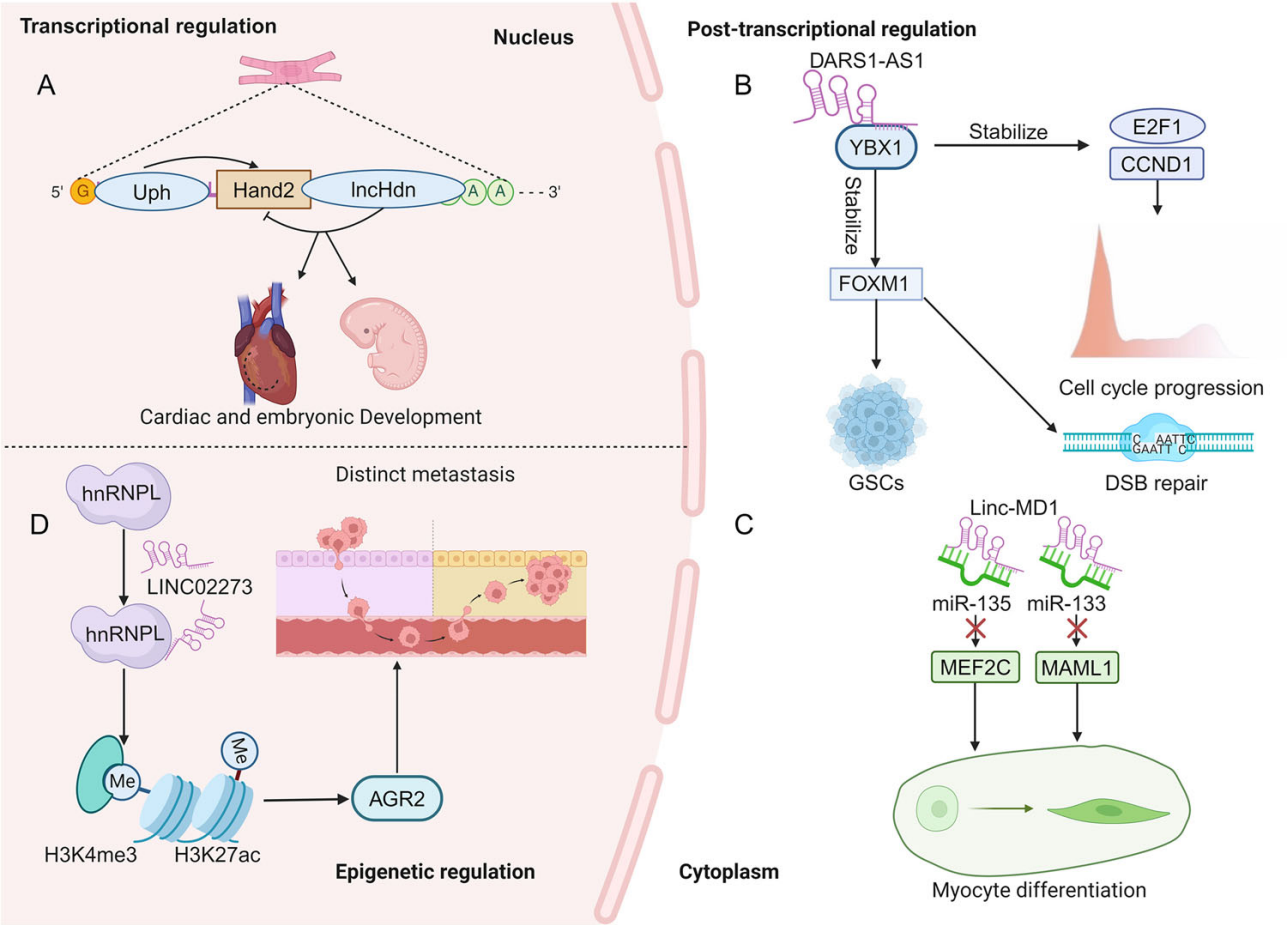
The molecular functions of lncRNAs. **A** lncRNA Hdn modulates cardiomyocyte and embryonic development via inhibition of Hand2 gene transcription. **B** lncRNA DARS1-AS1 interacts with YBX1 to stabilize FOXM1, E2F1 and CCND1, ultimately influencing cell cycle and GSCs renewal. **C** lncRNA-MD1 sequesters miR-135 and miR-133 to release MEF2C and MAML1 during myocyte differentiation. **D** LINC02273 and hnRNPL generate a complex to epigenetically promote AGR2 expression by increasing H3K4me3 and H3K27ac levels.

lncRNAs have gained increasing attention for their regulatory roles in various physiological and pathological processes. Although they are not translated into proteins, lncRNAs are crucial for gene regulation through interactions with DNA, RNA, and proteins at the transcriptional, post-transcriptional, and epigenetic levels [12]. (1) Transcriptional regulation: lncRNAs can act as enhancers or promoters to regulate the transcription of neighboring or distant protein-coding genes. They can recruit chromatin-modifying complexes to influence gene expression epigenetically [13]. For example, Ritter and colleagues revealed that lncRNA Hdn, which is activated in the early heart cells, transcriptionally suppresses the neighboring gene Hand2 (Fig. 1A) [14]. (2) Post-transcriptional regulation: lncRNAs can regulate mRNA stability, alternative splicing, and translation by interacting with RNA-binding proteins [15]. Zhang et al., observed that lncRNA DARS1-AS1 directly interacts with YBX1, impacting the expression of E2F1 and CCND1. Additionally, DARS1-AS1 stabilizes FOXM1 mRNA, ultimately promoting the malignant properties of glioblastoma stem cell-like cells (GSCs) (Fig. 1B) [16]. lncRNA may also act as competing endogenous RNAs (ceRNAs) to sequester microRNAs and modulate their activity [17]. This theory was first proposed in 2011 by Marcella Cesana et al., who found that lncRNA linc-MD1 regulates the formation of mouse myoblasts. Specifically, linc-MD1 prevents the suppression of muscle-specific genes MAML1 and MEF2C by sponging miRNA-135 and miRNA-133, ultimately promoting muscle differentiation (Fig. 1C) [18]. (3) Epigenetic regulation: lncRNAs can guide chromatin-modifying complexes to specific genomic loci, resulting in the alterations in histone modifications and DNA methylation patterns [7]. Xiu reported that LINC02273 contributes breast cancer tumor metastasis by interacting with hnRNPL, therefore epigenetically enhancing AGR2 expression through the augmentation of local H3K4me3 and H3K27ac levels (Fig. 1D) [19].

**Fig. 2:**
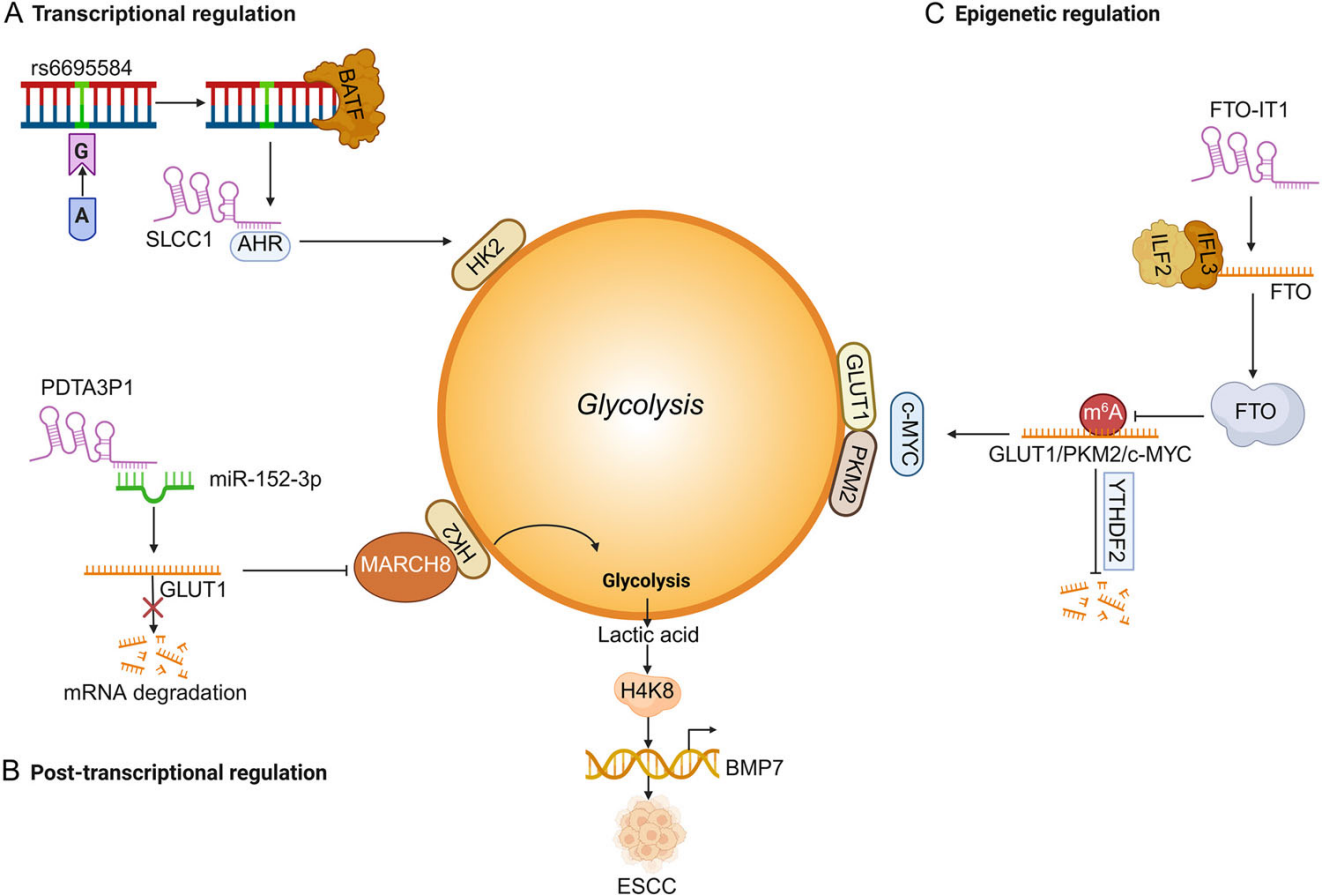
lncRNAs modulate glycolysis at different levels. **A** lncRNA SLCC1 transcriptional actives HK2 expression via the interaction with AHR in colon cancer. **B** lncRNA PDIA3P1 sponges miR-152-3p, leading to the upregulation of GLUT1 and lactate production in ESCC. **C** lncRNA FTO-IT1 reduces the m⁶A modification on GLUT1 and PKM2 to drive glycolysis and HCC growth.

These regulatory modalities collectively enable lncRNAs to fine-tune glycolysis. For example, the risk-associated SNP rs6695584 at the 1q41 locus creates a BATF-binding motif that enhances lncRNA-SLCC1 expression in colorectal cancer (CRC). Mechanistically, lncRNA-SLCC1 forms a complex with AHR to transcriptionally activate the glycolysis-promoting enzyme HK2, thereby driving glycolytic metabolism and accelerating CRC tumor growth (Fig. 2A) [20]. lncRNA PDIA3P1 serves as a ceRNA of miR-152-3p to prevent degradation of GLUT1, therefore promoting glycolysis, enhancing elevated lactate production and accelerating esophageal squamous cell carcinoma (ESCC) tumor progression (Fig. 2B) [21]. In addition, lncRNA FTO-IT1 enhances glycolysis through stabilizing FTO and reducing m⁶A modification on key glycolytic enzymes such as GLUT1 and PKM2, eventually promoting tumor progression (Fig. 2C) [22]. In the following section, we outline the fundamental roles of lncRNAs in glycolysis.

## lncRNAs regulate cancer glycolysis

Dysregulated lncRNAs modulate tumor glycolysis and reprogram cancer cell metabolism through diverse mechanisms (Table 1). Through modulation of glycolytic enzyme expression and activity, interaction with oncogenic signaling pathways, and reprogramming of the tumor microenvironment (TME), these lncRNAs promote the metabolic adaptations that support tumor growth and survival [23].

**Table 1:** The dysregulation of lncRNAs in cancer glycolysis.

LncRNA	Cancer type	Expression	Function and Mechanism	References
HOXC-AS3	BC	Up	Interacts with SIRT6 to prevent the inhibition of HIF1α, leading to reprogramming of metabolic pathways in breast cancer.	[137]
AC005392.2	CRC	Up	SOX2 overexpression enhances glycolysis and sustains VM formation via the transcriptional activation of lncRNA AC005392.2.	[138]
LINRIS	CRC	Up	Silencing of LINRIS attenuates IGF2BP2-MYC-mediated glycolysis.	[139]
MIR17HG	CRC	Up	Enforced MIR17HG expression is associated with poor survival, especially in patients with liver metastasis.	[140]
H19	GC	Up	Highly expressed in GC samples, and associated with tumor growth in vivo.	[141]
SNHG7	GC	Up	SNHG7 promotes cellular glycolysis and enhances cisplatin resistant.	[142]
HAGLR	GC	Up	Sponges miR-338-3p and enhances LDHA-modulated glycolysis pathway, therefore promoting cancer cell resistance to 5-Fu.	[143]
DLX6-AS1	GC	Up	Increases PDK1, enhances glycolysis and promotes tumor growth.	[144]
SH3BP5-AS1	HCC	Up	Boosts cell invasion, migration, and proliferation.	[145]
LINC00908	LUAD	Down	LINC00908 inhibits glycolysis by regulating the expression of the DDX54.	[146]
LncRNA-p23154	OSCC	Up	Upregulates GLUT1 expression, thereby promoting cellular glycolysis and accelerating tumor metastasis.	[147]
DLGAP1-AS2	OS	Up	Upregulates HK2 expression through sponging miR-451a to promote glycolysis.	[148]
LINC00160	PCa	Up	Mediates cell proliferation and metabolism by inhibiting RCAN1 expression.	[149]

*HOXC-AS3* HOXC cluster antisense RNA 3, *SOX2* SRY-box transcription factor 2, *VM* Vasculogenic mimicry, *LINRIS* long intergenic noncoding RNA for IGF2BP2 stability, *IGF2BP2* insulin like growth factor 2 mRNA binding protein 2, *MYC* MYC proto-oncogene, bHLH transcription factor, *MIR17HG* miR-17-92a-1 cluster host gene, *H19* H19 imprinted maternally expressed transcript, *LDHA* lactate dehydrogenase A, *SNHG7* small nucleolar RNA host gene 7, *HAGLR* HOXD antisense growth-associated long non-coding RNA, *DLX6-AS1* DLX6 antisense RNA 1, *PDK1* phosphoinositide-dependent protein kinase 1, *SH3BP5-AS1* SH3BP5 antisense RNA 1, *DDX54* DEAD-box helicase 54, DLGAP1-AS2, DLGAP1 antisense RNA 2, *HK2* hexokinase 2, *LINC00160* long intergenic non-protein coding RNA 160, *RCAN1* regulator of calcineurin 1, *BC* breast cancer, *CRC* colorectal cancer, *GC* gastric cancer, *HCC* hepatocellular carcinoma, *LUAD* lung adenocarcinoma, *OSCC* Oral Squamous Cell Carcinoma, *OS* osteosarcoma, *PCa* prostatic cancer.

### lncRNAs regulate the glycolysis process by affecting transporters

**Fig. 3:**
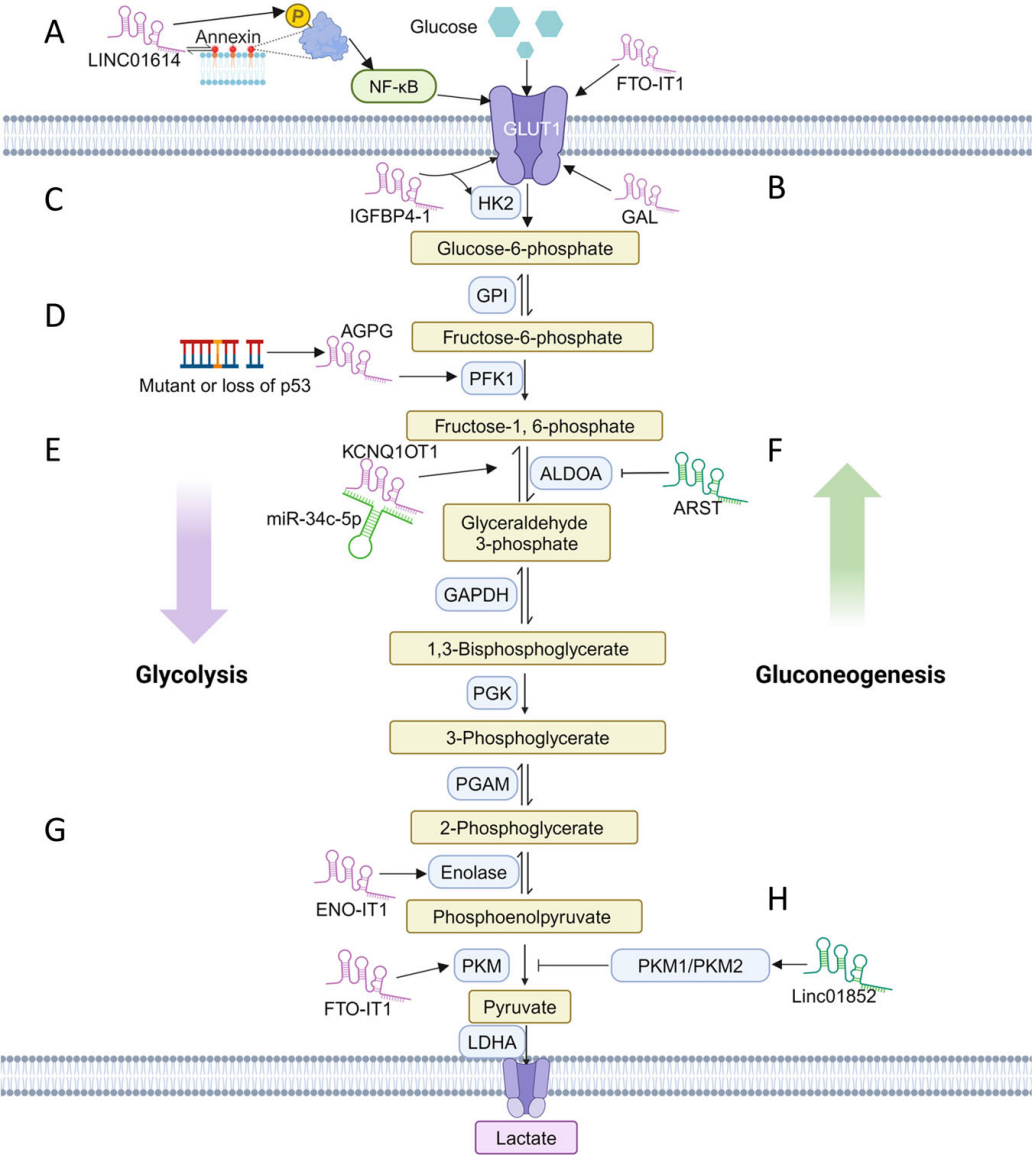
lncRNAs influence cancer glycolysis process by glycolytic and gluconeogenic enzymes. **A** Cancer-associated fibroblast–specific LINC01614 promotes glutamine uptake and metabolic support in lung adenocarcinoma via interaction with ANXA2, thereby driving tumor growth and progression. **B** lncRNA GAL stabilizes the GLUT1, thereby promoting glycolytic metabolism and metastatic progression of colon cancer. **C** lncRNA IGFBP4-1 overexpression enhances glycolysis to promote lung cancer cell proliferation and tumor progression. **D** lncRNA AGPG promotes tumor glycolytic reprogramming by stabilizing PFKFB3, thus enhancing glycolysis and driving cancer cell proliferation. **E** lncRNA KCNQ1OT1 promotes osteosarcoma growth by sponging miR-34c-5p, which upregulates ALDOA to enhance aerobic glycolysis. **F** lncRNA ARST suppresses glioma progression by disrupting ALDOA-mediated actin cytoskeleton integrity, consequently inhibiting tumor cell migration and invasion. **G** *F. nucleatum*–induced lncRNA ENO1-IT1 enhances ENO1-driven glycolysis to promote colorectal cancer growth and oncogenesis. **H** By blocking SRSF5-mediated PKM alternative splicing and decreasing PKM2 levels, LINC01852 impairs glycolysis, suppresses colorectal cancer progression and chemoresistance.

Glucose transporter (GLUT) proteins mediate cellular glucose uptake, sustaining energy production and biosynthesis essential for cancer growth, metastasis, and survival under stress [24, 25]. Experimental evidence suggests that lncRNAs can modulate the expression and activity of GLUT proteins [26]. For instance, Liu reported that LINC01614 directly interacts with ANXA2, promoting the phosphorylation of p65 at Ser276 and leading to NF-κB activation. Activated NF-κB subsequently transcriptionally regulates GLUTs SLC38A2 and SLC7A5, therefore contributing to the lung cancer development (Fig. 3A) [27]. In addition, lncRNA HOTAIR stimulates glucose uptake by the induction of GLUT1 and other glucose metabolism factors, such as PTEN and HIF-1α [28]. Zhang reported that lncRNA NEAT1_2 is significantly associated with poor survival of hepatocellular carcinoma (HCC). Mechanistically, mTOR represses NEAT1_2, releasing its binding proteins NONO and SFP1, which in turn facilitates GLUT1 splicing and enhance HCC progression [29]. Li et al., identified that lncRNA GAL promotes CRC metastasis by binding to GLUT1, inducing its SUMOylation and enhancing glucose uptake (Fig. 3B) [30]. lncRNA GAS6-AS1 inhibits tumorigenesis via suppressing E2F1-mediated GLUT1 expression in lung cancer [31]. Similarly, lncRNA AWPPH inhibits colon cancer cell proliferation by suppressing GLUT1 expression [32].

### lncRNAs and glycolytic enzymes

Glycolysis is series of metabolic reactions catalyzed by different enzymes, many of which are dysregulated in cancers [33]. Hereby, we summarize the latest research on the impact of glycolytic enzymes in cancer, with a particular focus on their regulation by lncRNAs (Table 2).

**Table 2:** LncRNAs regulate glycolytic enzymes.

LncRNA	Cancer type	Function and Mechanism	References
Oncogene			
YIYA	BC	Adjusts PFKFB3 to promote cancer glycolysis and tumor growth.	[45]
BCAR4	BC	Enhances the levels of HK2 and PFKFB3 in cancer.	[46]
DIO3OS	BCSC	Upregulates LDHA expression and activates glycolytic metabolism.	[150]
KCNQ1OT1	CRC	Interacts with HK2 to activate aerobic glycolysis and promote the occurrence of cancer.	[37]
SLCC1	CRC	Activates HK2 expression to drive glycolysis pathways and accelerate tumor growth.	[20]
BBOX1-AS1	CRC	Combines with PFK1 and promotes the proliferation and glycolysis of cancer.	[39]
MAFG-AS1	CRC	Upregulates PDK1 to promote glycolysis.	[40]
NONHSAG028908.3	CRC	Positively regulates ALDOA through sponge miR-34a-5p to promote the malignant activity of CRC.	[49]
ENO1-IT1	CRC	Activates ENO1 to promote tumor development.	[53]
CYTOR	CRC	Enhances the EMT of cancer through ENO2.	[151]
SNHG6	CRC	Targets PKM mRNA and enhances aerobic glycolysis in CRC cells.	[152]
AGPG	ESCC	Combines with PFKFB3 to activate glycolysis flux and promote cell cycle progression.	[43]
PTPRG-AS1	ESCC	Upregulates PDK1 expression and promotes glycolysis.	[153]
VAL	GC	Directly binds to PKM2 and enhances aerobic glycolysis in cancer cells.	[58]
ARSR	Glioma	Enhances aerobic glycolysis through the STAT3-HK2 axis to promote the development of glioma.	[154]
ZFPM2-AS1	HCC	Interacts with PKM2 to promote glycolysis and tumor development.	[60]
HULC	HCC	Increases the phosphorylation of LDHA and PKM2 to promote glycolysis.	[155]
IGFBP4-1	LC	Upregulates the expression of ATP and metabolic enzymes including HK2 to promote tumor growth.	[36]
CCHE1	Melanoma	Binds with LDHA and promotes the glycolytic flux of melanoma cells.	[156]
LINC00930	NPC	Activates PFKFB3 to promote glycolysis flux and cell cycle progression	[44]
CYB561-5	NSCLC	Promotes the expression of HK2 and PFK1 to alter cancer metabolic reprogramming.	[41]
LINC00973	Pan cancer	Enhances LDHA activity and promotes glycolysis.	[64]
UROD	PC	Accelerates glycolysis of cancer cells through PKM/ENO1 mediated pathway.	[59]
GLTC	PTC	Targets the succinylation and enzymatic activity of LDHA to promote cancer progression.	[63]
Tumor suppressor			
LINC00926	BC	Inhibits the Warburg effect and tumor growth via PGK1.	[157]
LINC01852	CRC	Regulates the alternative splicing of PKM and inhibits tumor growth.	[61]
ARST	Glioma	Directly binds to ALDOA to inhibit tumor growth.	[52]
PTCSC3	PTC	Interacts with PGK1 to inhibit cancer aerobic glycolysis.	[158]

*YIYA* long intergenic non-protein coding RNA 538, *BCAR4* breast cancer anti-estrogen resistance 4, *DIO3OS* DIO3 opposite strand upstream RNA, *KCNQ1OT1* KCNQ1 opposite strand, *SLCC1* a single nucleotide polymorphism mediated lncRNA, *BBOX1-AS1* BBOX1 antisense RNA 1, *MAFG-AS1* MAFG antisense RNA 1, *NONHSAG028908.3* long intergenic non-protein coding RNA 1123, *ENO1-IT1* enolase1-intronic transcript 1, *CYTOR* cytoskeleton regulator RNA, *EMT* epithelial-mesenchymal transition, *SNHG6* small nucleolar RNA host gene 6, *AGPG* actin gamma, *PTPRG-AS1* PTPRG antisense RNA 1, *VAL* vimentin-associated lncRNA, *ARSR* activated in renal cell carcinoma with sunitinib resistance, *STAT3* signal transducer and activator of transcription 3, *ZFPM2-AS1* ZFPM2 antisense RNA 1, *HULC* hepatocellular carcinoma up-regulated long non-coding RNA, *IGFBP4-1* insulin-like growth factor binding protein 4-1, *CCHE1* cervical carcinoma high expressed 1, *LINC00930* long intergenic non-protein coding RNA 930, *CYB561-5* antisense to TANC2, *LINC00973* Long intergenic non-protein coding RNA 973, *UROD* Uroporphyrinogen decarboxylase, *GLTC* Glycolysis associated regulator of LDHA post-transcriptional modification, *LINC00926* Long intergenic non-protein coding RNA 926, *LINC01852* Long intergenic non-protein coding RNA 1852, *ARST* long intergenic non-protein coding RNA 632, *PTCSC3* Papillary thyroid carcinoma susceptibility candidate 3, *BC* breast cancer, *BCSC* breast cancer stem cells, *CRC* colorectal cancer, *ESCC* esophageal squamous cell carcinoma, *GC* gastric cancer, *HCC* hepatocellular carcinoma, *LC* lung cancer, *NPC* nasopharyngeal carcinoma, *NSCLC* non-small cell lung cancer, *PC* pancreatic cancer, *PTC* papillary thyroid carcinoma.

#### HK2

HK catalyzes the first irreversible step in glucose metabolism, converting glucose into glucose-6-phosphate. This step effectively capture glucose within cells, enhancing glucose utilization, ATP synthesis, glucose storage, NADH pool enrichment and protein glycosylation [34]. Among the hexokinase isozymes, HK2 is highly expressed in tumor samples and correlate with poor pathological outcome [35]. Yang reported that lncRNA IGFBP4 promotes cell proliferation and tumor growth both in vitro and in vivo. Mechanistically, enforced IGFBP4 upregulates HK2 along with other metabolic enzymes such as GLUT1, Aldolase A (ALDOA), PGK1, pyruvate kinase M2 (PKM2), phosphoinositide-dependent kinase (PDK1), thereby facilitating ATP production in lung cancer. However, these effects could be reversed by 2-DG (a specific inhibitor of glycolysis), rhodamine 123 (Rho123, an inhibitor of mitochondrial oxidative phosphorylation), or a combination of 2-DG and Rho123, respectively (Fig. 3C) [36]. In addition, lncRNA KCNQ1OT1 interacts with HK2 to promote CRC carcinogenesis by activating aerobic glycolysis [37]. lncRNA NEAT1_2 recruits KDM5B to repress RRAD through histone modification of H3K4me3, thereby activating transcriptional factor EHF and promoting HK2 expression in papillary thyroid carcinoma (PTC) [38]. Yan and colleagues identified that lncRNA SLCC1 interacts with AHR to increase HK2 expression and glycolysis in CRC [20].

#### PFK1

Phosphofructokinase-1 (PFK1), the second rate-limiting enzyme in glycolysis, catalyzes the conversion of fructose 6-phosphate (F-6-P) and ATP to fructose 1,6-bisphosphate (F-1,6-BP) and ADP [35]. Wang reported that lncRNA BBOX1-AS1 binds and enhances the ability of PFK1, therefore promoting radiation resistance in CRC [39]. lncRNA MAFG-AS1 facilitates glycolysis by inhibiting miRNA-147b and activating NDUFA4, causing an upregulation of PFK1 in CRC [40]. lncRNA CYB561-5 interacts with protein basigin (Bsg) to upregulate HK2 and PFK1 expression in non-small cell lung cancer (NSCLC) [41].

#### PFKFB3

Fructose-2,6-bisphosphate (F-2,6-BP), a product of F-6-P catalyzed by PFKFB3, is considered to be the most potent allosteric activator of PFK1, boosting its activity even in the presence of ATP [42]. Liu et al., found that loss or mutation of p53 drives lncRNA AGPG, which interacts with and stabilizes PFKFB3, thereby activating glycolytic flux and promoting cell cycle progression in ESCC (Fig. 3D) [43]. Similarly, LINC00930 is overexpressed in nasopharyngeal carcinoma, where it promotes glycolytic flux and cell cycle progression by activating H3K4 trimethylation and H3K9 acetylation of PFKFB3 through interactions with RBBP5 and GCN5 [44]. In breast cancer, lncRNA YIYA interacts with CDK6, enhancing CDK6-dependent PFKBP3 phosphorylation, resulting in the promotion of glycolysis, cell proliferation, and tumor growth [45]. lncRNA BCAR4, a downstream target of Yes-associated protein (YAP), coordinates with hedgehog signaling to strengthen HK2 and PFKFB3 level in breast cancer [46].

#### ALDOA

ALDOA is a key enzyme in glycolysis that catalyzes the conversion of fructose-1,6-bisphosphate (F-1,6-BP) into glyceraldehyde-3-phosphate (G-3-P) and dihydroxyacetone phosphate in glycolysis [47]. Shen et al., showed that lncRNA KCNQ1OT1 functions as a ceRNA by sponging miRNA-34c-5p, thereby reactivating ALDOA in osteosarcoma (Fig. 3E) [48]. Additionally, lncRNA NONHSAG028908.3 targets miRNA-34a-5p, and counteract the inhibitory effect of miRNA-34a-5p on ALDOA [49]. In gastric cancer, lncRNA PSMA3-AS1 represses miRNA-329-3p, leading to increased ALDOA level, while knockdown of miRNA-329-3p or overexpression of ALDOA can partially attenuate tumor-suppressive effect of PSMA3-AS1 knockdown [50]. lncRNA ZNF674-AS1 interacts with ALDOA and promotes its binding to ATP6V1B2, a non-catalytic subunit of v-ATPase, thereby activating AMPK signaling in granulosa cell [51]. Interestingly, lncRNA also present a non-metabolic activity by interacting with ALDOA. lncRNA ARST represses cell growth and migration in gliomas by preventing the interaction between ALDOA and F-actin, resulting in the rapid, cofilin-dependent disassembly of F-actin stress fibers (Fig. 3F) [52].

#### Enolase

Enolase is a key enzyme in the ninth step of glycolytic pathway, driving the conversion of 2-phosphoglycerate (2-PG) to phosphoenolpyruvate (PEP). There are three enolase isoenzymes, each composes of different subunits: ENO1 (α-enolase), ENO2 (γ-enolase) and ENO3 (β-enolase) [11]. Hong recently presented that Fusobacterium nucleatum induces lncRNA ENO1-IT1 by upregulating the transcriptional factor SP1. Elevated ENO1-IT1 serve as scaffold for the activation of KAT7 histone acetyltransferase, leading to the upregulation of downstream genes, including ENO1, which in turn promotes glucose metabolism and CRC tumorigenesis (Fig. 3G) [53]. AL355338 is an oncogenic lncRNA upregulated in NSCLC that binds to and stabilizes ENO1, therefore enhancing glycolysis and driving tumor progression via the EGFR/AKT signaling pathway [54].

#### PKM

Pyruvate kinase M (PKM) exists in two isoforms, PKM1 and PKM2, both serving as key glycolytic enzymes that catalyze the final step of glycolysis, converting phosphoenolpyruvate into pyruvate and generating ATP [55]. PKM1 is key for energy production in tissues with high energy demands, while PKM2 is important for supporting anabolic processes in proliferating cells, particularly in cancer. PKM2 has become a significant focus in cancer research and therapeutic development [56, 57]. lncRNAs modulate PKM2 stability via inhibiting its ubiquitination during tumor metabolic reprogramming. For example, lncRNA VAL interacts with PKM2, abrogating the PKM2-Parkin interaction, therefore preventing PKM2 degradation and aerobic glycolysis in gastric cancer [58]. lncRNA UROD binds to and prevents the ubiquitination of ENO1 and PKM, thereby stabilizing these glycolytic enzymes to enhance glycolysis, proliferation, and migration in pancreatic cancer cells [59]. lncRNA ZFPM2-AS1 inhibits M1 polarization (IL-12, TNF-α, IFN-γ), promotes M2 infiltration (IL-10 and IL-4) through interaction with PKM2 in HCC [60]. LINC1852 interacts with SRSF5, enhancing its degradation, which increases PKM1/PKM2 ratio and represses aerobic glycolysis, tumor growth and chemoresistance (Fig. 3H) [61].

#### LDHA

Lactate dehydrogenase A (LDHA) is a key glycolytic enzyme that catalyzes the conversion of pyruvate to lactate while regenerating NAD⁺, which is essential to sustain continuous glycolytic flux [62]. lncRNA GLTC enhances LDHA succinylation and enzymatic activity to drive glycolysis, thereby promoting papillary thyroid cancer progression and conferring resistance to radioiodine therapy [63]. LINC00973 directly binds to LDHA, amplifying its enzymatic activity and thereby enhancing aerobic glycolysis [64]. Activation of the chemokine receptor CCR7 induces expression of the lnc-Dpf3 in dendritic cells, where it directly binds HIF-1α and represses its transcriptional activity. Silenced HIF-1α could reverse the lnc-Dpf3 induced LDHA expression, thereby restraining dendritic cell migration and modulating immune responses [65].

In summary, these findings highlight the intricate interplay between lncRNAs and glycolytic enzymes in cancer, offering insights into novel therapeutic targets for cancer treatment.

## lncRNAs regulate glycolysis through transcription factors

Transcription factors are essential for glycolysis [66–68]. lncRNAs can shape the Warburg effect by regulating transcription factors that control glycolytic enzymes (Table 3).

**Table 3:** LncRNAs regulate transcription factors.

LncRNA	Cancer type	Function and mechanism	References
Oncogene			
HISLA	BC	Disrupts the PHD2/HIF1α interaction and enhances HIF1α expression to promote tumor glycolysis.	[78]
HIFAL	BC	Forms a positive feedback loop with HIF1α to maintain the glycolysis pathway.	[159]
LINC00525	CRC	Activates HIF1α to enhance hypoxia-induced glycolysis.	[160]
GLCC1	GC	Enhances MYC/IGF2BP1 interaction and regulates cancer cell migration and invasion.	[71]
SLC2A1-DT	HCC	Promotes glycolysis and tumorigenesis through m⁶A modification mediated MYC.	[74]
FTO-IT1	HCC	Upregulates the expression of MYC to enhance glycolysis.	[22]
FAM83A-AS1	LUAD	Promotes cancer cell proliferation and stemness through the HIF1α/glycolysis axis.	[161]
AC016727.1	NSCLC	Regulates the expression of HIF1α and promotes tumor growth.	[162]
LINC01123	NSCLC	Controls proliferation and aerobic glycolysis through the positive feedback loop of MYC.	[163]
PCGEM1	PCa	Regulates tumor metabolism via activating MYC.	[75]
MIF	Pan-cancer	Inhibits aerobic glycolysis and tumorigenesis via downregulation of MYC.	[164]
lincRNA-p21	Pan-cancer	Interacts with HIF1α to promote glycolysis and tumor growth under hypoxic conditions.	[165]
NEAT1_2	PTC	NEAT1_2/RRAD/EHF complex promotes cancer glycolysis.	[38]
Tumor suppressor			
PWRN1	HCC	Reduces LDHA in MYC and inhibits the growth of tumor cells.	[72]
IDH1-AS1	Pan cancer	Downregulates HIF1α to inhibit glycolysis and tumor growth.	[166]
DPF3	Pan cancer	Directly binds to HIF1α and inhibits DC glycolytic metabolism.	[167]

*HISLA* HIF1A stabilizing long noncoding RNA, *PHD2* known as EGLN1, egl-9 family hypoxia inducible factor 1, *HIFAL* HIF1A anti-sense lncRNA, *LINC00525* long intergenic non-protein coding RNA 525, *GLCC1* known as FARP1, FERM/ARH/RhoGEF and pleckstrin domain protein 1, *MYC* Myc proto-oncogene and bHLH transcription factor, *SLC2A1-DT* SLC2A1 divergent transcript, *FTO-IT1* FTO Intronic Transcript 1, *FAM83A-AS1* FAM83A antisense RNA 1, *AC016727.1* a lncRNA located on chromosome 2, *LINC01123* long intergenic non-protein coding RNA 1123, *PCGEM1* prostate-specific transcript, *MIF* macrophage migration inhibitory factor, lincRNA-p21, a previously identified p53-inducible lncRNA, NEAT1_2 nuclear paraspeckle assembly transcript 1_2, *RRAD* ras related glycolysis inhibitor and calcium channel regulator, *EHF* ETS homologous factor, *PWRN1* Prader-Willi region non-protein coding RNA 1, *IDH1-AS1* IDH1 antisense RNA 1, *DPF3* double PHD fingers 3, *DC* dendritic cells, *BC* breast cancer, *CRC* colorectal cancer, *GC* gastric cancer, *HCC* hepatocellular carcinoma, *LUAD* lung adenocarcinoma, *NSCLC* non-small cell lung cancer, *PCa* prostatic cancer, *DC* dendritic cell.

### MYC

**Fig. 4:**
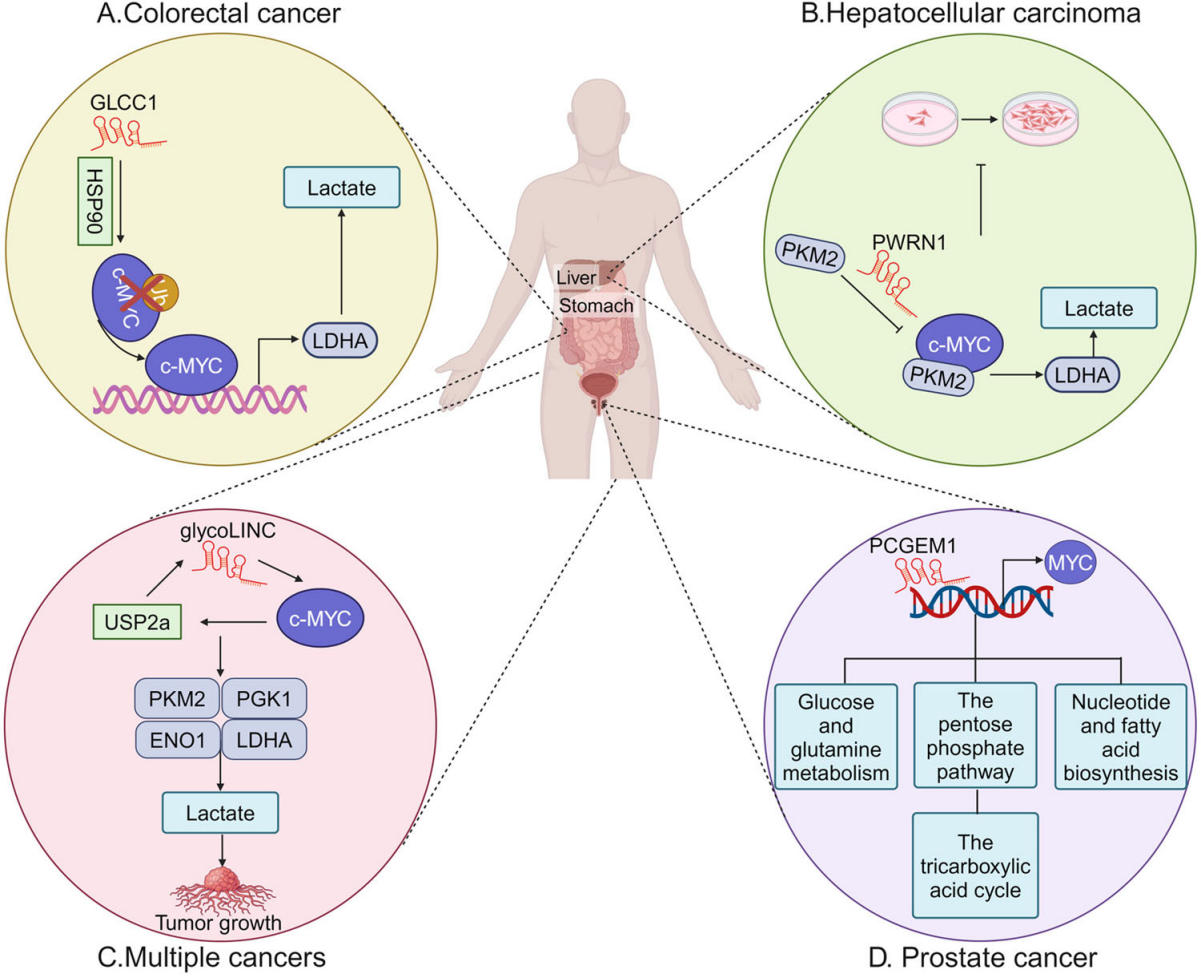
lncRNAs regulate glycolysis through MYC. **A** In colorectal cancer, lncRNA GLCC1 stabilizes MYC via directly interacting with HSP90, leading to the activation of LDHA and promotion of lactate. **B** lncRNA PWRN1 interacts with glycolytic enzyme PKM2, thereby reducing the expression of MYC and LDHA, and inhibiting HCC proliferation. **C** MYC-responsive lncRNA glycoLINC, facilitates the interaction between key glycolytic enzymes. **D** lncRNA PCGEM1 promotes MYC activity to enhance the glycolysis and glutamine metabolism, the pentose phosphate pathway, nucleotide and fatty acid biosynthesis, and the tricarboxylic acid cycle.

MYC, a key driver of glycolysis, is commonly dysregulated across multiple cancers [66, 69]. In CRC, lncRNA GLCC1 promotes tumorigenesis and glycolysis by binding HSP90 to stabilize MYC, thereby enhancing transcription of target genes such as LDHA (Fig. 4A) [70]. Similarly, GLCC1 upregulation enhances the interaction between IGF2BP1 and MYC, further strengthening MYC stability and driving gastric cancer development [71]. Conversely, lncRNA PWRN1 suppresses cell proliferation by inhibiting PKM2 activity and nuclear translocation, thereby reducing MYC-driven LDHA expression and glycolysis in HCC (Fig. 4B) [72]. Zhu reported that a MYC-responsive lncRNA called gLINC enhances cancer glycolysis and tumor formation by assembling glycolytic enzymes such as ENO1 and PGK1, PKM2, and LDHA (Fig. 4C) [73].

lncRNAs may also regulate MYC level. Zeng recently revealed that lncRNA SLC2A1-DT interacts with YWHAZ to stabilize the β-catenin, eventually improving MYC expression. In turn, MYC enhances the stability of SLC2A1-DT in a YTHDF1-dependent manner, suggesting SLC2A1-DT and MYC may form a positive feedback loop in HCC [74]. Similarly, lncRNA PCGEM1 generates a double-activated loop with MYC, contributing glycolysis, glutamine metabolism, pentose phosphate pathway, nucleotide and fatty acid biosynthesis, and the tricarboxylic acid cycle in prostate tumor (Fig. 4D) [75].

### HIF1α

**Fig. 5:**
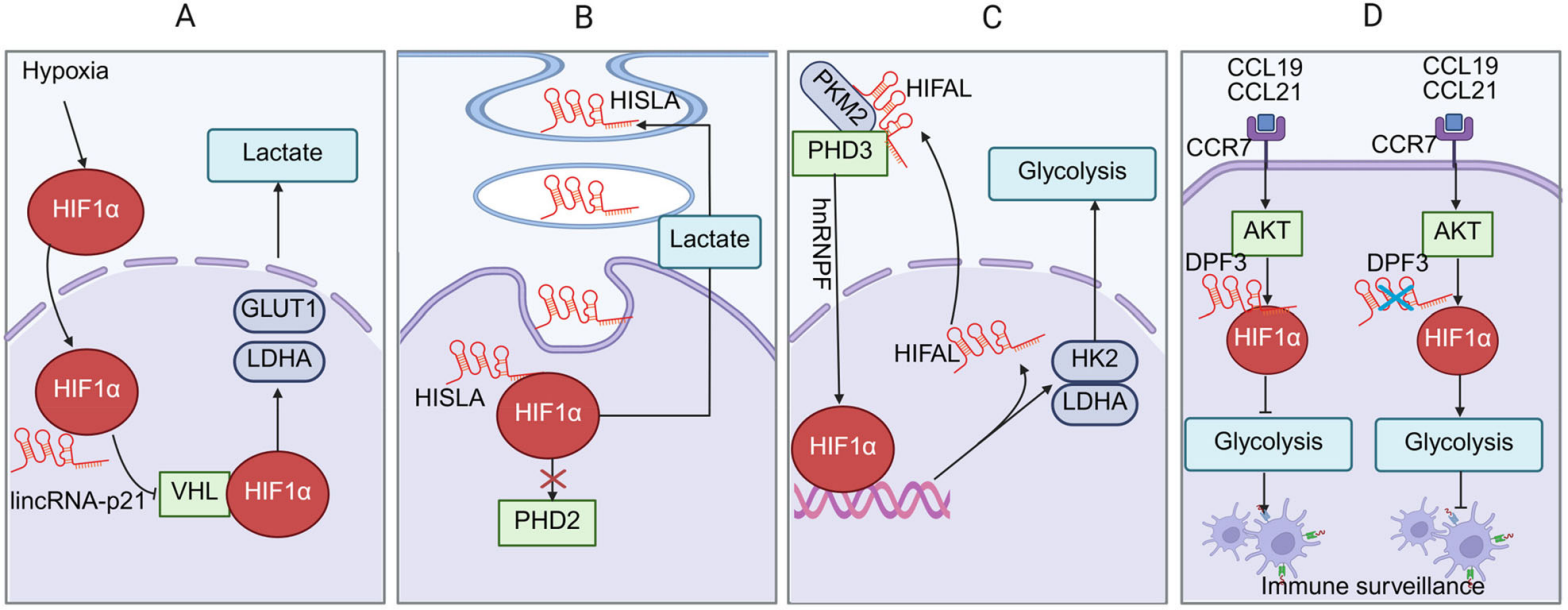
lncRNAs regulate glycolysis through HIF1A. **A** lincRNA-p21 prevents VHL-mediated HIF-1α ubiquitination and causes HIF1α accumulation, resulting in promotion of glycolysis and tumor growth. **B** lncRNA HISLA disrupts PHD2/HIF1α interaction, resulting in increased HIF1α expression and enhanced glycolysis in TAMs. **C** lncRNA HIFAL promotes glycolysis by interacting with the PHD3/PKM2 complex, which binds hnRNPF and activates HIF1α-dependent HK2, LDHA. **D** lncRNA DPF3 reverses HIF1α-responsive glycolysis and cell migration.

Hypoxia-inducible factor 1α (HIF1α) is a master transcriptional regulator activated under low-oxygen conditions, where it orchestrates the cellular adaptive response by promoting glycolysis and suppressing oxidative phosphorylation [76]. Yang reported that lincRNA-p21 interacts with HIF1α, disrupting HIF1α/VHL axis and leading to accumulating of HIF1α, which subsequently promotes glycolysis and tumor growth under hypoxia condition (Fig. 5A) [77]. HIF-1α-stabilized lncRNA HISLA disrupts the PHD2/HIF1α interaction, leading to increased HIF1α expression and enhanced glycolysis in tumor-associated macrophages (TAMs) (Fig. 5B) [78]. Moreover, lncRNA HIFAL produces a positive feedback loop with HIF1α to maintain glycolysis pathway. Mechanistically, HIFAL induces propyl hydroxylation of PKM2 by interacting with PHD3, facilitating the nuclear translocation of the PHD3/PKM2 complex to activate HIF1α-dependent glycolysis (Fig. 5C) [79].

Conversely, lncRNA IDH1-AS1 has been shown to inhibit tumor growth by suppressing glycolysis through downregulation of HIF1α [80]. Liu et al., showed that lncRNA DPF3 attenuates this glycolytic metabolism and migratory capacity by directly binding to HIF1α, thereby preventing HIF1α-dependent glycolytic gene expression, such as LDHA (Fig. 5D) [65].

In summary, lncRNAs are pivotal regulators of glucose metabolism in cancer, exerting their influence through direct interactions with transcription factors such as MYC and HIF1α, thereby modulating the expression of key glycolytic enzymes and sustaining tumor metabolic reprogramming.

## lncRNAs regulate transduction pathways

lncRNAs exert pivotal roles in regulating glucose metabolism through various signaling pathways in cancer (Table 4).

**Table 4:** LncRNAs regulate signaling pathways.

LncRNA	Cancer type	Regulatory mechanism	References
Oncogene			
RBM5-AS1	CRC	Interacts with β-catenin and enhances WNT signaling.	[92]
SNHG3	CRPC	Sequesters miR-199a-5p and enhances PKM2 expression to promote proliferation and tumor glycolysis.	[168]
LINC00470	GBM	Activates the AKT signaling pathway to promote cancer progression.	[84]
HOTAIR	HCC	Promotes glycolysis.	[85]
MALAT1	HCC	Activates the mTORC1-4EBP1 axis and promotes tumor metastasis.	[86]
Ftx	HCC	Activates the PPARγ pathway to promote tumor growth.	[169]
AP000695.2	LUAD	Regulates the miR-335-3p/TEAD1 axis to promote cancer glycolysis.	[170]
AL355338	NSCLC	Interacts with ENO1 to activate EGFR and AKT in cancer.	[54]
AC020978	NSCLC	Activates PKM2/HIF-1α axis and promotes cancer cell proliferation and glycolytic metabolism.	[171]
LINC00857	OC	Regulates Hippo signaling to promote tumor progression and glycolysis.	[172]
HOTAIRM1	OS	Promotes glycolysis and tumor growth induced by miR-664b-3p/Rheb/mTOR pathway.	[173]
HCG18	OS	Regulates the miR-365a-3p/PGK1 axis to enhance aerobic glycolysis.	[174]
HIF1A-AS1	PC	Promotes glycolysis in pancreatic cancer through AKT/YB1/HIF1α pathway.	[175]
LINC00941	PDAC	Activates Hippo pathway to enhance glycolysis.	[94]
GHET1	TNBC	Activates the Hippo/YAP signaling pathway and promotes tumor progression.	[176]
Tumor suppressor			
DUXAP8	AML	Inhibits glycolysis through the Wnt/β-catenin pathway.	[177]
FGF13-AS1	BC	Inhibits cancer glycolysis through the FGF13-AS1/IGF2BPs/MYC feedback loop.	[178]
MACC1-AS1	GC	Inhibits AMPK and promotes tumor growth.	[89]
LINC01554	HCC	Inhibits the AKT/mTOR signaling pathway and eliminates glycolysis.	[83]
LCAL1	LC	Reduces AMPK signaling and increases cell proliferation and aerobic glycolysis.	[88]
EPB41L4A-AS1	Pan cancer	Knockdown of EPB41L4A-AS1 expression to trigger Warburg effect.	[96]

*RBM5-AS1* RBM5 antisense RNA 1, *WNT* wingless-type MMTV integration site family, *SNHG3* small nucleolar RNA host gene 3, *LINC00470* long intergenic non-protein coding RNA 470, *AKT* knows as PKB, protein kinase B, *HOTAIR* HOX transcript antisense RNA, *mTOR* mechanistic target of rapamycin kinase, *MALAT1* metastasis associated lung adenocarcinoma transcript 1, *mTORC1* mammalian target of rapamycin complex 1, *4EBP* eukaryotic translation initiation factor 4E binding protein 3, *Ftx* FTX transcript and XIST regulator, *PPARγ* peroxisome proliferator-activated receptor γ, *AP000695.2* PPP1R3B divergent transcript, *TEAD1* TEA domain transcription factor 1, *AL355338* an upregulated glycolysis-associated lncRNA, *EGFR* epidermal growth factor receptor, *AC020978* an lncRNA located on human chromosome 16, *LINC00857* long intergenic non-protein coding RNA 857, *HOTAIRM1* HOXA transcript antisense RNA, myeloid-specific 1, *Rheb* Ras homolog, mTORC1 binding, *HCG18* HLA complex group 18, *HIF1A-AS1* HIF1A antisense RNA 1, *YB1* Y-box binding protein 1, *LINC00941* long intergenic non-protein coding RNA 941, *GHET1* gastric carcinoma proliferation enhancing transcript 1, *YAP* yes-associated protein, *DUXAP8* double homeobox A pseudogene 8, *FGF13-AS1* FGF13 antisense RNA 1, *IGF2BPs* insulin-like growth factor 2 mRNA binding proteins, *MACC1-AS1* MACC1 antisense RNA 1, *LINC01554* long intergenic non-protein coding RNA 1554, *LCAL1* lung cancer associated lncRNA 1, *EPB41L4A-AS1* EPB41L4A antisense RNA 1, *CRC* colorectal cancer, *CRPC* castration-resistant prostate cancer, *GBM* glioblastoma multiforme, *HCC* hepatocellular carcinoma, *LUAD* lung adenocarcinoma, *NSCLC* non-small cell lung cancer, *OC* ovarian cancer, *OS* osteosarcoma, *PC* pancreatic cancer, *PDAC* pancreatic ductal adenocarcinoma, *TNBC* triple-negative breast cancer. *AML* acute myeloid leukemia, *BC* breast cancer, *GC* gastric cancer, *LC* lung cancer.

### PI3K/AKT/mTOR pathway

**Fig. 6:**
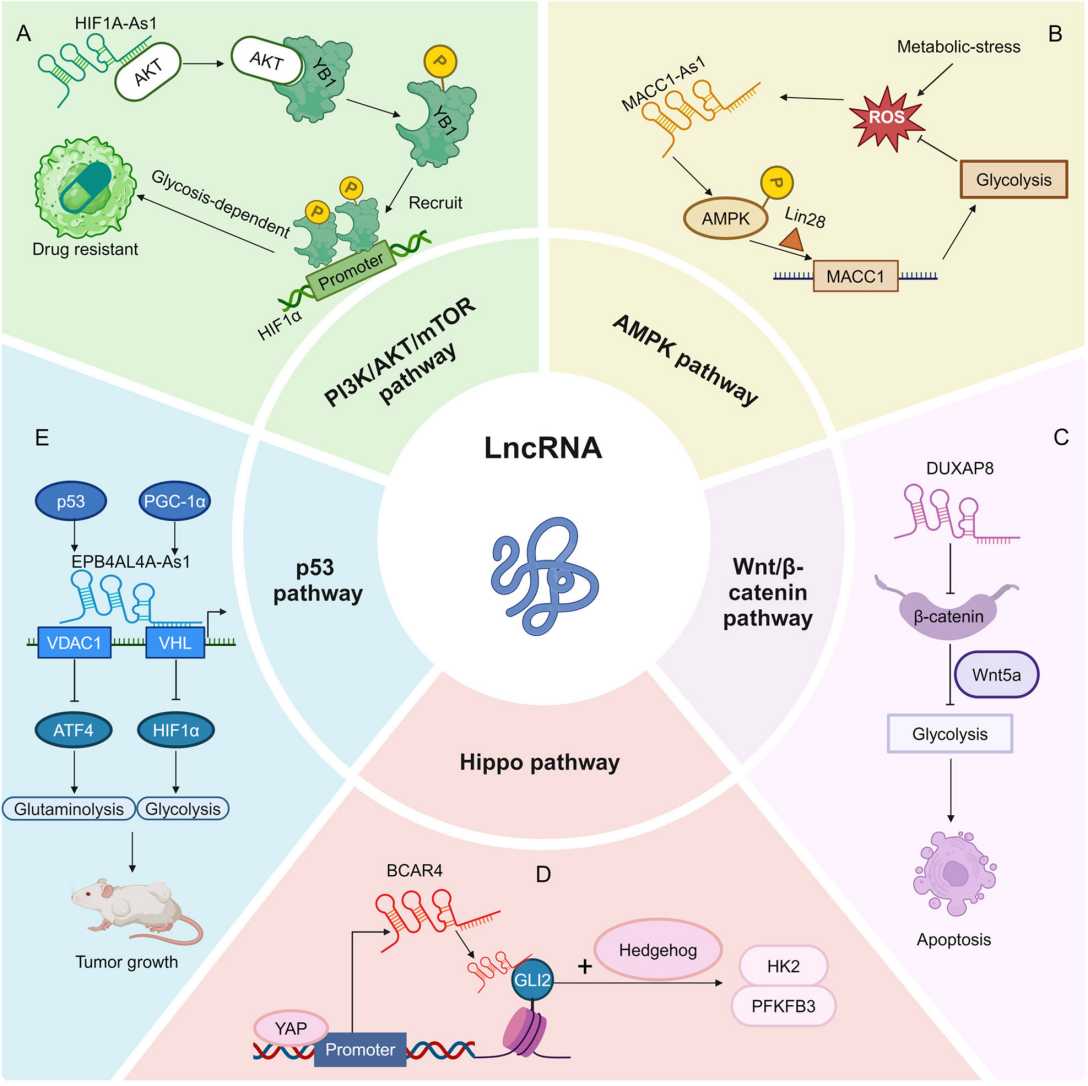
lncRNAs regulate transduction pathways. **A** lncRNA HIF1A-AS1 promotes glycolysis and drug resistance via AKT signaling. **B** lncRNA MACC1-AS1 stabilizes MACC1 level and promotes glycolysis via phosphorylation of AMPK/Lin28. **C** lncRNA DUXAP8 inhibits glycolysis and induces apoptosis of AML cells by inhibiting the Wnt/β-catenin pathway in acute myeloid leukemia. **D** YAP-activated lncRNA BCAR4 coordinates with the Hedgehog signaling pathway to enhance transcription of glycolytic activators such as HK2 and PFKFB3. **E** p53-induced lncRNA EPB41L4A-AS1 regulates VDAC1 and VHL expression through interaction with HDAC2 to, regulating glutaminolysis and glycolysis.

One important pathway through which lncRNAs influence glucose metabolism is the PI3K/AKT/mTOR pathway [81]. lncRNAs can reshape the PI3K/AKT/mTOR pathway to influence glucose metabolism. Xu and colleagues reported that lncRNA HIF1A-AS1 binds to AKT, enhances AKT/YB1 interaction, and facilitates the phosphorylation of YB1 (pYB1), which is then recruited to the HIF1α promoter to activate its transcription and drive the glycolysis-dependent drug resistance in pancreatic cancer (Fig. 6A) [82]. LINC01554 exerts tumor-suppressive functions by promoting ubiquitin-mediated degradation of PKM2 and inhibiting AKT and mTOR phosphorylation in HCC [83]. LINC00470 interacts with FUS to enhance its phosphorylation, which promotes AKT nuclear translocation and reduces ubiquitinated-modulated degradation of HK1, thereby sustaining glycolysis glioblastoma [84]. HOTAIR activates mTOR signaling, therefore leading to increased glucose uptake and glycolysis in HCC [85]. In addition, MALAT1 promotes cell growth and tumor metastasis by controlling TCF7L2 expression via mTOR-mediated TCF7L2 translation, thereby facilitating aerobic glycolysis and repressing gluconeogenesis in HCC [86].

### AMPK pathway

The AMP-activated protein kinase (AMPK) pathway functions as a cellular energy sensor that regulates glucose metabolism in response to energy fluctuations [87]. lncRNAs have been increasingly recognized as modulators of AMPK-driven glucose metabolism in cancer. For instance, lncRNA LCAL1 increases cell proliferation and aerobic glycolysis by repressing of AMPK signaling in lung cancer [88]. lncRNA MACC1-AS1, which is highly expressed in gastric cancer, promotes cell viability by accelerating glucose uptake and increasing ATP and lactate production. MACC1-AS1 upregulates key glycolytic enzymes, including GLUT1, HK2, and LDH, thereby reinforcing the glycolytic phenotype of cancer cells via promotion of AMPK phosphorylation (Fig. 6B) [89].

### Wnt/β-catenin pathway

The Wnt/β-catenin signaling pathway is intricately involved in various aspects of cancer biology [90]. lncRNA DUXAP8 inhibits glycolysis via suppression of the Wnt/β-catenin pathway in acute myeloid leukemia (AML) (Fig. 6C) [91]. lncRNA LUST, a nuclear-retained lncRNA, directly interacts with β-catenin, enhancing its occupation at promoter regions of WNT targets genes, thereby influencing cancer metabolism [92].

### Hippo pathway

The hippo pathway is a crucial signaling pathway involved in glycolysis and cellular metabolism [93]. In triple-negative breast cancer, lncRNA GHET enhances hypoxia-induced Hippo/YAP pathway, facilitating the translocation of YAP into nuclear and activating glycolysis-related target genes [93]. LINC00941 drives the PP2A-mediated dephosphorylation of MST1 to activate the Hippo pathway and enhance glycolysis in pancreatic ductal adenocarcinoma (PDAC) [94]. In addition, YAP transcriptionally activates lncRNA BCAR4, which coordinates with the Hippo signaling to enhance the transcription of glycolysis-related genes, such as HK2 and PFKFB3 (Fig. 6D) [46].

### p53 pathway

The p53 pathway is a critical cellular mechanism responsible for maintaining genomic stability and prevents tumorigenesis [95]. lncRNA AGPG, a transcription target of p53, is essential for cell proliferation and metabolism remodeling in ESCC via enhancing the stability of PFKFB3 [43]. The p53-induced lncRNA EPB41L4A-AS1 is dysregulated in multiple cancers and interacts with HDAC2 to modulate the VHL/HIF-1α pathway. Its knockdown drives HDAC2 translocation to the nucleoplasm, increasing occupancy on VHL and VDAC1 promoters, thereby enhancing glycolysis and glutaminolysis to support tumor survival and growth (Fig. 6E) [96].

## lncRNA regulates protein synthesis

Protein synthesis and energy metabolism are intimately intertwined processes in cellular biology [97, 98]. lncRNAs can influence protein synthesis through various mechanisms at both the transcriptional and post-transcriptional levels, thereby affecting cellular energy demands and metabolic pathways.

Firstly, lncRNAs can influence pre-mRNAs splicing, resulting in the production of different mRNA isoforms from a single gene. These isoforms may differ in their translation efficiency or encode proteins with distinct functions, thereby impacting protein synthesis both qualitatively and quantitatively. For example, MALAT1 stabilizes the interaction between two splicing factors, polypyrimidine tract-binding protein 1 (PTBP1) and PTB-associated SF (PSF), forming a functional module that affects a network of alternative splicing events [99].

Secondly, lncRNAs can regulate the initiation of protein synthesis by modulating ribosome access to mRNA [7]. lncRNA FIRRE interacts with PTBP1, triggering its translocation from nucleus to cytoplasm, which in turn, enhances the mRNA stability of BECA1 [100]. Additionally, LOC101928222 stabilizes HMGCS2 mRNA through IGF2BP1 in an m^6^A-dependent pathway, leading to increased cholesterol synthesis and promoting CRC development [101].

Thirdly, lncRNAs can also bind to translation factors, modifying their activity and influencing the efficiency of translation. Hu observed that lncRNAs such as SNHG12, FTX, SNHG5, and ZNFX1-AS1 interact with eukaryotic initiation factor EIF4E, a critical component of the translation initiation machinery, which in turn contributes MYC translation in mantle cell lymphoma cells [102]. lncRNA LCETRL3/LCETRL4 stabilize TDP43 or EIF2S1, leading to increased levels of NOTCH1 or phosphorylated PDK1, thereby activating AKT signaling and promoting resistance to EGFR-tyrosine kinase inhibitors (TKIs) in NSCLC [103]. In summary, these mechanisms illustrate the versatile roles of lncRNAs in protein synthesis, influencing key processes such as alternative splicing, translation initiation, and the modulation of translation factors.

## lncRNA-encoded micropeptides

Recent studies have uncovered that lncRNA-encoded micropeptides are critical for cancer development and progression [104]. These lncRNA-encoded micropeptides often interact with metabolic enzymes, modulating their activity and influencing cancer metabolism [105–107]. A micropeptide encoded by lncRNA HOXB-AS3 inhibits glucose metabolic reprogramming in CRC by competitively antagonizing hnRNP A1 to reduce the levels of PKM. This regulatory mechanism counteracts the Warburg effect, thereby limiting the reliance of colon cancer cells on glycolysis for energy production [108]. In glioblastoma, a micropeptide encoded by LINC02381 has been identified through machine learning approaches as a regulator of ferroptosis via the glucose transporter SLC2A10 [109]. The micropeptide NEMEP, encoded by lncRNA Gm11549 and regulated by Nodal signaling, supports mesendoderm differentiation by binding GLUT1/GLUT3 to enhance glucose uptake for metabolic demands [110]. Overall, lncRNA-encoded micropeptides represent a novel layer of regulation in cancer metabolism.

## lncRNAs regulate tumor microenvironment

The TME is a complex network of cancer cells, stromal cells, immune infiltrates, extracellular matrix (ECM), and signaling molecules. lncRNAs influence various cellular processes within the TME, which include immune responses, fibroblast activation, ECM remodeling, and the metabolic adaptation of tumor cells [111].

### lncRNA and tumor-associated macrophages

**Fig. 7:**
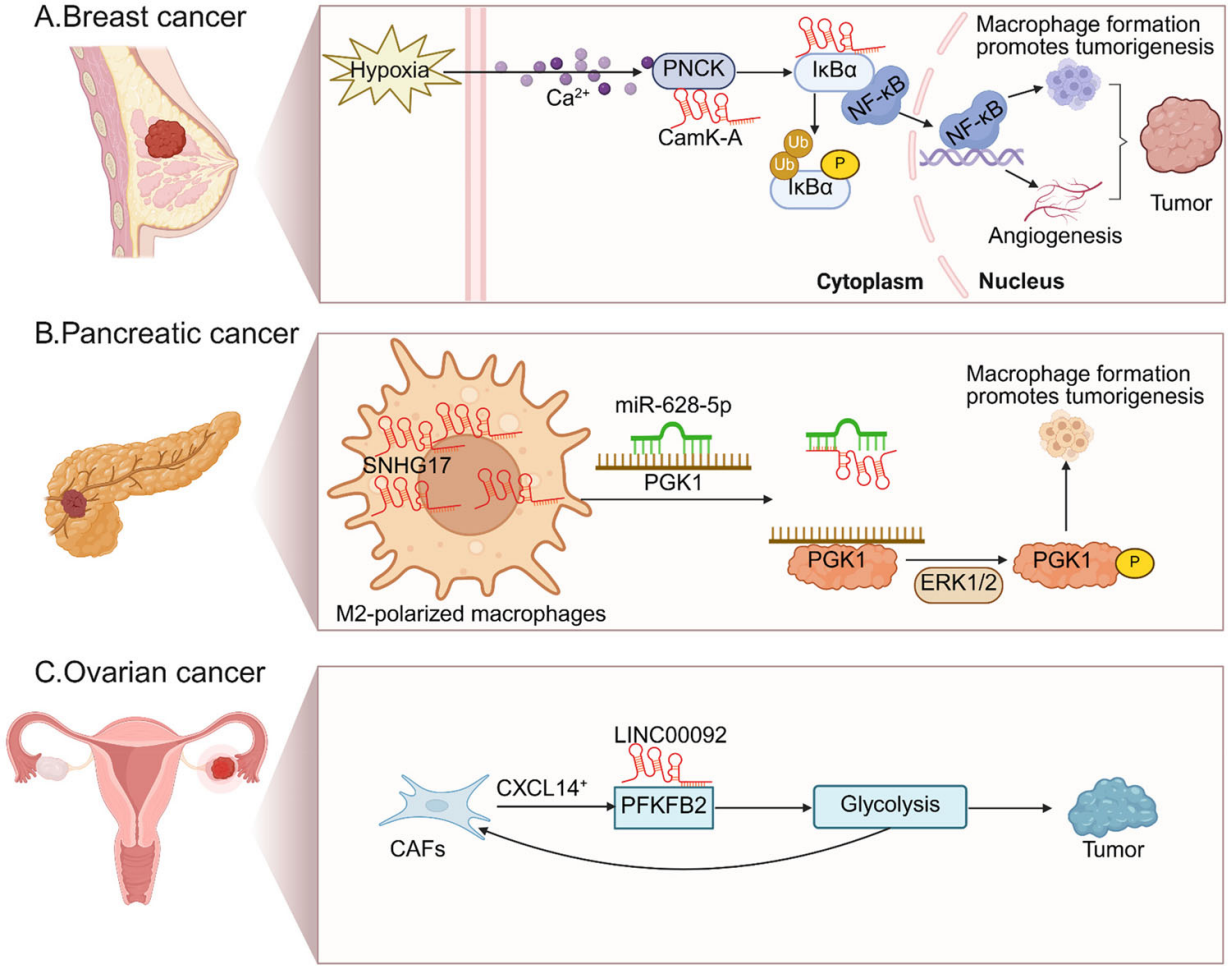
lncRNAs regulates tumor microenvironment. **A** lncRNA CamK-A affects TME by Ca²⁺/calmodulin-dependent phosphorylation of IκBα and activation of NF-κB in breast cancer. **B** SNHG17 sponges miR-628-5p and therefore releasing PGK1 to enhance ERK1/2 signaling. **C** LINC00092 interacts with PFKFB2 to enhance glycolysis and promote tumor progression.

TAMs are a key component of TME and are frequently associated with poor prognosis and resistance to therapy, including immunotherapy [112]. lncRNA CamK-A is upregulated in multiple cancers, where it activates Ca²⁺-PNCK–NF-κB signaling to remodel the TME through angiogenesis and macrophage recruitment. By reinforcing aerobic glycolysis via enhanced extracellular acidification rate (ECAR) and GLUT3 expression, CamK-A promotes tumor progression, while its inhibition suppresses growth, highlighting its potential as a biomarker and therapeutic target (Fig. 7A) [113]. SNHG17 is highly enriched in TAMs, where it promotes anaerobic glycolysis by sponging miR-628-5p to upregulate PGK1 expression. It also binds PGK1 to recruit ERK1/2, enhancing PGK1 phosphorylation at the T168A site, which reprograms its activity to drive pro-tumorigenic (M2-like) macrophage polarization and thereby sustaining pancreatic cancer progression (Fig. 7B) [114]. TAMs promote HCC malignancy by transferring the exosomal lncMMPA into tumor cells, where it acts as a microRNA sponge for miR-548s to upregulate ALDH1A3, and thereby enhancing glycolytic metabolism and tumor growth [115]. TAMs also secrete TNFα to induce lncRNA MALR in esophageal cancer cells, where MALR binds and stabilizes ILF3. This promotes ILF3 liquid–liquid phase separation and activates HIF-1α signaling, consequently facilitating aerobic glycolytic activity and tumor progression [116].

### lncRNA and cancer-associated fibroblasts

Cancer-associated fibroblasts (CAFs) are a dominant and heterogeneous cell population within the TME. They actively promote tumor cell invasion and metastasis, support immune evasion, stimulate angiogenesis, and reinforce therapeutic resistance [117, 118]. CAFs-derived chemokine CXCL14 induces the expression of LINC00092, which binds PFKFB2 to enhance glycolysis, establishing a metabolic feedback loop that drives metastasis and correlates with poor prognosis (Fig. 7C) [119]. The lncRNA H19/miR-675-5p/PFKFB3 axis drives glycolysis in CAFs and enhances proliferation, migration, and tumor growth in oral squamous cell carcinoma (OSCC) [120]. lncRNA TUG1, which is enriched in CAFs secrete exosomes, sponges miR-524-5p to increase the level of SIX1. This activation leads to enhanced glycolytic metabolism by accelerating glucose uptake, lactate production, LDH activity, and ATP levels, consequently promoting cell migration and invasion in liver cancer [121].

### lncRNAs and other immune cells

Liu identified that CCR7 stimulation enhances the HIF-1α level in DCs, leading to metabolic reprogramming toward glycolysis for DC migration. However, ectopic lnc-Dpf3 could interact with HIF-1α and suppress its activation of glycolytic gene LDHA, thus inhibiting DC glycolytic metabolism and migratory capacity [65].

In summary, lncRNAs regulate glycolysis within the TME through intricate interactions that modulate both signaling pathways and the expression of key glycolytic enzymes.

## Clinical applications

lncRNAs can serve as biomarkers for the detection and prognosis of various cancers (Table 5). For example, elevated expression of H19 is associated with poor prognosis in various cancers (e.g., colorectal, liver, and breast cancer) [122]. The presence of H19 in tumor tissues or body fluids could serve as a non-invasive diagnostic biomarker for early detection or monitoring of disease progression [123]. In addition, circulating lncRNAs detected in plasma, serum, or exosomes could complement established biomarkers such as LDHA or PD-L1 [64, 124]. For example, elevated levels of HOTAIR in cancer cells are associated with poor prognosis and aggressive tumor stages. Its interaction with glycolytic enzymes makes it a potential biomarker for assessing glycolytic reprogramming in tumors [125, 126].

**Table 5:** Clinical applications of lncRNAs in cancer.

LncRNA	Cancer type	Clinical application	Function/Role in cancer
HOTAIR	Breast, liver, gastric, colorectal cancer	Diagnostic, Prognostic, Therapeutic	Upregulated in various cancers; enhances aerobic glycolysis; promotes metastasis and poor prognosis; potential therapeutic target.
MALAT1	Lung, breast, glioma, colon, prostate cancer	Diagnostic, Prognostic, Therapeutic	Associated with metastasis; regulates metastasis-related genes; stabilizes glycolytic enzymes; potential prognostic biomarker.
UCA1	Bladder, gastric, liver, ovarian, pancreatic cancer	Diagnostic, Prognostic	Overexpressed in cancers; sustains EMT signaling.
H19	Breast, liver, gastric, colon cancer	Prognostic	Overexpression links to poor prognosis, metastasis, and chemoresistance.
PVT1	Ovarian, bladder, pancreatic, gastric cancer	Prognostic, Therapeutic	Associated with poor prognosis and chemoresistance; inhibits ferroptosis; potential target for therapy.
LINC00152	Liver, breast, gastric cancer	Prognostic	Promotes tumor progression, cell invasion and metastasis; associated with poor survival outcomes.
MEG3	Various cancer	Therapeutic (Tumor-suppressive)	Tumor-suppressive; restoration of MEG3 reverses oncogenic phenotypes.
CCAT2	Colon, gastric, lung, pancreatic cancer	Diagnostic, Prognostic	Associated with tumor development; a potential biomarker for early diagnosis.
XIST	Breast, colon, lung, ovarian cancer	Prognostic	Involved in X-chromosome inactivation; regulates key tumor-suppressive pathways.
Linc-ROR	Various cancers, especially in cancer stem cells	Therapeutic, Prognostic	Regulates cancer stem cell properties and glycolysis; potential therapeutic target.
TUG1	Lung, prostate, breast cancer	Prognostic	Upregulated in various cancers; associated with EMT; promotes cell proliferation and tumor progression.
RMRP	lung and bladder cancer	Prognostic	Overexpressed in cancer samples; regulates cell cycle and apoptosis; associated with poor prognosis.
AFAP1-AS1	Colon, gastric, breast, pancreatic cancer	Prognostic	Overexpressed in multiple cancers; induces therapeutic resistance; affects macrophage polarization, enhances cell migration and invasion.

RNA therapy, particularly the utilization of Antisense Oligonucleotides (ASOs), represents a promising approach in the treatment of various diseases, including cancer [127]. ASOs are short, synthetic strands of nucleotides that bind to specific RNA sequences, leading to degradation of target gene through RNase H-mediated cleavage [128]. A breakthrough in the treatment of Duchenne muscular dystrophy has been achieved through the application of ASOs in promoting exon skipping [129]. In addition, systemic administration of ASOs has effectively reduced the expression of CUG (exp) RNA and MALAT1, resorting normal physiological, histopathologic and transcriptomic features in the myotonic dystrophy type 1 [130]. In cancer, ASOs against HOTAIR have shown the ability to modulate the TME, thereby inhibiting tumor growth [131]. Similarly, in lung cancer, ASO targeting lncRNA HIF1A-As2 significantly inhibits tumor growth in a patient-derived xenograft (PDX) model [132]. Targeting glycolysis-related lncRNAs might help sensitize tumors to chemotherapy by reducing the metabolic adaptation that allows cancer cells to survive.

Despite the promising potential of lncRNAs in regulating glycolysis and tumor metabolism, several challenges remain [133, 134]. [1] Delivery methods: Effectively delivering lncRNA-targeting therapies to tumor cells remains a key hurdle, especially in solid tumors with complex microenvironments. [2] Off-target effects: Off-target effects are a critical issue, as lncRNAs often have pleiotropic functions and sequence similarity with other transcripts, increasing the risk of unintended gene regulation. In addition, synthetic nucleic acids can activate innate immune responses through recognition by pattern recognition receptors such as Toll-like receptors, leading to inflammation or toxicity. To mitigate these risks, strategies such as chemical backbone modifications (e.g., 2′-O-methyl, phosphorothioate, locked nucleic acids), optimized sequence design, and careful preclinical validation are being actively pursued. [3] Stability: Circulating lncRNAs often display variable stability due to nuclease degradation, and standardization of extraction and quantification methods remain lacking. [4] Limitations and Contradictions: Many investigations are based on small patient cohorts or single cancer cell lines, restricting the generalizability of their conclusions. Mechanistic insights are often derived from in vitro assays without validation in animal models or large, independent clinical datasets, which raises concerns about their translational relevance. Moreover, contradictory findings have emerged for certain lncRNAs. For instance, NEAT1 promotes breast cancer progression by forms a scaffold bridge for the assembly of PGK1/PGAM1/ENO1 complexes, yet other studies describe tumor-suppressive functions of NEAT1 in different contexts [135, 136]. These limitations underscore the need for validation across diverse cohorts, multi-omics integration, and standardized methodologies to resolve conflicting evidence and establish robust conclusions regarding lncRNA function in cancer metabolism.

## Unanswered questions

Despite rapid advances, many important questions remain unresolved in the study of lncRNAs and cancer metabolism. It is unclear that how lncRNAs exert cell-type–specific effects across tumor, stromal, and immune compartments within the TME. Allelic variants and alternative isoforms of lncRNAs may differentially influence glycolytic reprogramming and therapeutic responses. Another unresolved issue is how lncRNAs integrate with other regulatory layers, such as post-translational modifications and metabolic flux at the systems level.

Several emerging technologies provide promising avenues to tackle these gaps. Single-cell multi-omics and spatial transcriptomics are revealing striking heterogeneity of lncRNA expression across tumor subsets and niches, offering unprecedented resolution into context-dependent functions. Spatial transcriptomics is revolutionizing our understanding of lncRNA function in personalized medicine by enabling the high-resolution mapping of gene expression in the tissue context. Unlike bulk or even single-cell RNA sequencing, spatial transcriptomics preserves the spatial localization of lncRNA expression within the TME, revealing how lncRNAs regulate region-specific processes such as hypoxia, immune evasion, or stromal remodeling. Spatial metabolomics techniques enable direct mapping of lncRNA-associated metabolic changes in situ. Tools like 10x Genomics Visium is now being used to build lncRNA-based spatial atlases, allowing clinicians to identify region-specific therapeutic vulnerabilities and stratify patients based on the spatial expression of lncRNA biomarkers. In addition, high-throughput CRISPR-based functional screens now allow systematic dissection of lncRNA roles in glycolysis and cancer progression. Furthermore, artificial intelligence and machine learning approaches are increasingly applied to integrate multi-omics datasets and predict regulatory lncRNA networks. Together, these technologies are poised to reshape our understanding of lncRNAs and accelerate their application in precision oncology.

## Conclusion and prospects

lncRNAs are now recognized as key regulators of cancer metabolism, driving glucose reprogramming, tumor growth, and metastasis. Their dysregulation makes them promising biomarkers and therapeutic targets, with potential for non-invasive diagnostics and personalized therapies. Beyond modulating glycolytic enzymes, lncRNAs can also encode micropeptides that directly regulate metabolism, adding complexity to cancer biology. RNA-based therapies, including ASOs, show early promise in reversing drug resistance, though challenges such as delivery efficiency and in vivo stability remain. Continued research into lncRNAs as diagnostic and therapeutic tools will advance precision oncology and improve patient outcomes.

## Data Availability

Data sharing is not applicable in this article as no new data was created or analyzed in this study.
